# Teamwork and relational infrastructure: a qualitative study of modern UK general practice

**DOI:** 10.3399/BJGP.2025.0603

**Published:** 2026-04-08

**Authors:** Francesca H Dakin, Ninna Meier, Emma Ladds, Sietse Wieringa, Joseph Wherton, Sarah Rybczynska-Bunt, Asli Kalin, Lucy Moore, Trisha Greenhalgh

**Affiliations:** 1 Nuffield Department of Primary Care Health Sciences, University of Oxford, Oxford, UK; 2 Department of Sociology and Social Work, Aalborg University, Aalborg, Denmark; 3 Centre for Health and Social Care Innovation, Faculty of Health, University of Plymouth, Plymouth, UK

**Keywords:** general practice, practice organisation, qualitative research, social sciences, teamwork

## Abstract

**Background:**

Clinical and support staff in modern general practice must work across in-person and digital modalities to deliver high-quality, safe care in a context of high workload, constant change, and intermittent crisis. Navigating this environment is cognitively and emotionally demanding, and requires complex teamwork. Staff morale may often be low, and staff turnover high.

**Aim:**

To understand how the context of modern UK general practice affects staff wellbeing and teamwork, and to develop our understanding of how to improve these aspects of work culture.

**Design and setting:**

This paper reports on a multi-sited case study of 10 general practices across England, Wales, and Scotland.

**Method:**

Multiple qualitative methods, including ethnographic observations, interviews, and focus groups, were used to develop case studies. First, we conducted in-depth longitudinal case studies of two practices focused on developing theory, which we cross-compared with eight other, more focused case studies. Our analysis was informed by theories from organisational research, including psychological safety, relational coordination, and attentional infrastructure.

**Results:**

Staff wellbeing and effective teamwork depended on positive team relations. Practices in which such relations were valued and nurtured (that is, those with a strong relational infrastructure) appeared to have stronger team identities, better coordination of work tasks, and higher overall staff wellbeing than those in which team relations were not actively nurtured. Staff relations were built and sustained through various individual actions and organisational routines.

**Conclusion:**

This study has identified several elements of 'relational infrastructure' with the potential to improve team relations, communication, and coordination, which may also enhance practices' resilience to withstand change and crises.

## How this fits in

General practice staff must now work across digital and in-person modalities in demanding conditions requiring complex coordination, often contributing to poor staff wellbeing and high turnovers. This article explored how positive team relations and ‘relational infrastructure’ affect staff wellbeing and work in modern general practice, drawing on qualitative case studies. In this sample, stronger relational infrastructure was associated with improved coordination, team identity, and staff wellbeing. Guidance is offered on what individual actions and organisational routines may help develop stronger relational infrastructure.

## Introduction

Modern general practice work requires staff to organise, support, and deliver patient care in the context of constant change and co-occurring crisis events, while working across digital and physical spaces. Crises may be abrupt (a sudden significant change to the established ‘normal’) or cumulative (something that has built up over time).^1^ In recent years, UK general practices have faced multiple crises, both abrupt (such as the COVID-19 pandemic, winter pressures) and cumulative (such as workforce shortages and resource constraints). Alongside this, these organisations have worked on integrating technology (for example, remote access, changes to practice software, remote consultations, the NHS app) and implementing incremental changes to work roles, processes, and routines. The most significant abrupt crisis has been the COVID-19 pandemic, in response to which UK general practices faced a mandate to introduce ‘remote by default’ care to enable digital and telephone access, triage, and consultation solutions, alongside digitalised and automated practice tasks and workflows.^2,3^ It is within this context that this multi-sited qualitative case study was conducted.

General practices delivered digitalisation alongside COVID-19-related workload changes, a growing backlog of non-COVID-19 clinical needs,^4,5^ a reduced workforce because of temporary sickness or burnout,^6–10^ and subsequently a post-lockdown increase in patient contact,^11^ as well as a loss of public confidence in general practice following adverse press.^12^ This was all amid severe workforce shortages across clinical and support roles (particularly in socioeconomically deprived areas),^4,13^ an associated rise in new organisational roles (such as allied professionals and physician associates),^13,14^ increasingly complex and fragmented workflows,^15–17^ and a growing supply and demand gap.^13^

Previous publications have described some of the effects of these changes and crises on staff work and wellbeing, such as technology-induced stress and suffering,^17^ strain on team relationships,^17^ moral injury in staff,^18^ and a higher burden to support patients in navigating increasingly complex access environments.^19,20^ However, we still know relatively little about how general practice teams maintain effective working relationships and wellbeing under the chronic pressures described above.

This article outlines the impact of such change and crisis events on general practice staff’s wellbeing and it identifies the role of relational infrastructure in enabling teams to overcome these incidents, thereby improving wellbeing and efficiency at work. We recognise that the term ‘wellbeing’ is nebulous and imprecise, with precise meaning hard to pin down even within the NHS’s own health and wellbeing framework.^21^ In this paper, we apply it broadly to refer to the mental and physical health of staff, reflecting its colloquial use in our dataset.

To define relational infrastructure, we draw specifically on three theoretical perspectives from the organisation studies literature: Gittell’s relational coordination theory,^22^ Edmondson’s psychological safety,^23,24^ and Bartel and Rockmann’s attentional infrastructures,^25^ as defined in Box 1 .^22,23,25–30^ These perspectives helped us to understand how change and crisis contexts challenge staff wellbeing and organisational efficiency, and what actions and rotines contribute to improvements therein.

**Box 1.** Defining the theoretical framework

Theory	Description
Relational coordination	Denotes the communication, trust, and shared routines that allow teams to function effectively, particularly under pressure Defined as *‘a mutually reinforcing process of communicating and relating for the purpose of task integration’* ^27^ This process happens via frequent, timely, accurate, and problem-solving communication through relationships of shared goals, shared knowledge, and mutual respect^27,28^ It is supported by various mechanisms and structures, such as boundary-spanner roles, team meetings, relational leadership, shared accountability, and shared conflict resolution,^22,26^ and is considered critical to achieving interdependent work, particularly in a context of unpredictability^28^
Psychological safety	Refers to *‘people’s perceptions of the consequences of taking interpersonal risks in a particular context such as a workplace’* ^23^ At an individual level, it enables personal engagement, creativity, and knowledge sharing at work, especially in relation to uncertain tasks; it is a precondition for speaking up^29^ At the organisational level, it is associated with improved organisational performance and learning from failure,^30^ a critical capability in a workplace facing frequent change and uncertainty
Attentional infrastructure	Focuses on the attention paid by organisations to interpersonal relationships among staff, with a focus on leadership, recognising that this *‘continually shapes and is shaped by the quality of relationships that emerge at the unit level’* ^25^ There are three archetypes of attentional infrastructure: relational advocacy*,* whereby leaders advocate for the value of interpersonal relationships and take active steps to nurture those relationships;^25^ relational antipathy, whereby leaders explicitly devalue the strategic importance of interpersonal relationships;^25^ and relational indifference, whereby leaders lack substantive attention in either direction, and may exhibit contradictions between espoused values and action^25^

Relational coordination and psychological safety have, together, generated useful insights in other healthcare workforce contexts such as resuscitation,^31^ trauma care,^32,33^ emergency and critical care departments, and during the acute phase of the COVID-19 pandemic.^18^ Psychological safety has gained popularity since its introduction to the field of organisational studies in a study of hospital teams,^24^ having been applied in primary and secondary care contexts in the UK and internationally.^17,34–36^ Although it is seen to improve patient safety and staff outcomes, there have been challenges in measuring the effectiveness of interventions aimed at improving psychological safety because of hard-to-define outcome measures,^37^ and likely because of the inherently contextual and experiential nature of the concept. By incorporating it into our conceptualisation of ‘relational infrastructure’, we aim to clarify how to identify and establish psychological safety in real-world settings.

Relational coordination, although developed in other organisational contexts, has been applied in research on team training in various secondary healthcare settings to improve medical teams’ ability to relate and communicate effectively,^26,31–33,38^ and in medical education to enhance students’ readiness to engage in such teamwork.^39^ Relational coordination has, to our knowledge, not yet been applied to the study of UK general practice teamwork beyond our own work.^17^

Attentional infrastructure, which has not yet been applied to healthcare contexts, focuses on the attention paid by organisations to interpersonal relationships among staff with a particular focus on leadership.^25^ We use this concept in its authors’ intended capacity; to generate *‘new insight on the pre-crisis period; specifically, preventive measures that may reduce vulnerability’*.^25^ Each of these theories provides excellent framing concepts for relational infrastructure in this setting, particularly given the established importance of (clinician–patient) relationships in improving patient care, experience, and workload in general practice,^40–43^ and the unique working style of general practice, which is characterised by smaller teams and increasingly hybridised and digitised working environments.^17,20^

## Method

### Design and setting

This study was part of a broader project called ‘Remote by Default 2: The new normal?’ (RBD2). The study aimed to inform the provision of high-quality, safe, and equitable care in UK general practice, following the mandate for remote primary care to be offered ‘by default’.^44^ The broader study’s design is described in its protocol and summative findings publications.^44–46^ In brief, RBD2 was a multi-sited case study utilising an adapted researcher-in-residence model to collect qualitative data from 12 general practices across England, Wales, and Scotland using observations and interviews.^47^ Data were collected between September 2021 and April 2024.

This article reports the findings of a substudy of 10 general practices, focusing on staff teamwork during change and crisis. Data were collected between June 2022 and April 2024 to ensure data saturation and sufficient engagement with the daily work of these general practices over an extended period. The aims were to describe how the context of modern UK general practice affects staff wellbeing and teamwork, and to develop our understanding of how to improve these aspects of work culture.

We conducted a multi-sited case study involving two longitudinal sites (at which the first author was the embedded researcher) and eight comparative sites (at which the third, fourth, fifth, sixth, seventh, and eighth authors were the embedded researchers). The 10 practices were sampled to represent maximum diversity across practice location, list size, population demographics, and digital maturity.^44,45^ The number of included practices was determined to balance representativeness of the heterogeneity of these features across UK general practice with the availability of researchers and the practicality of access.

**Figure 1.**
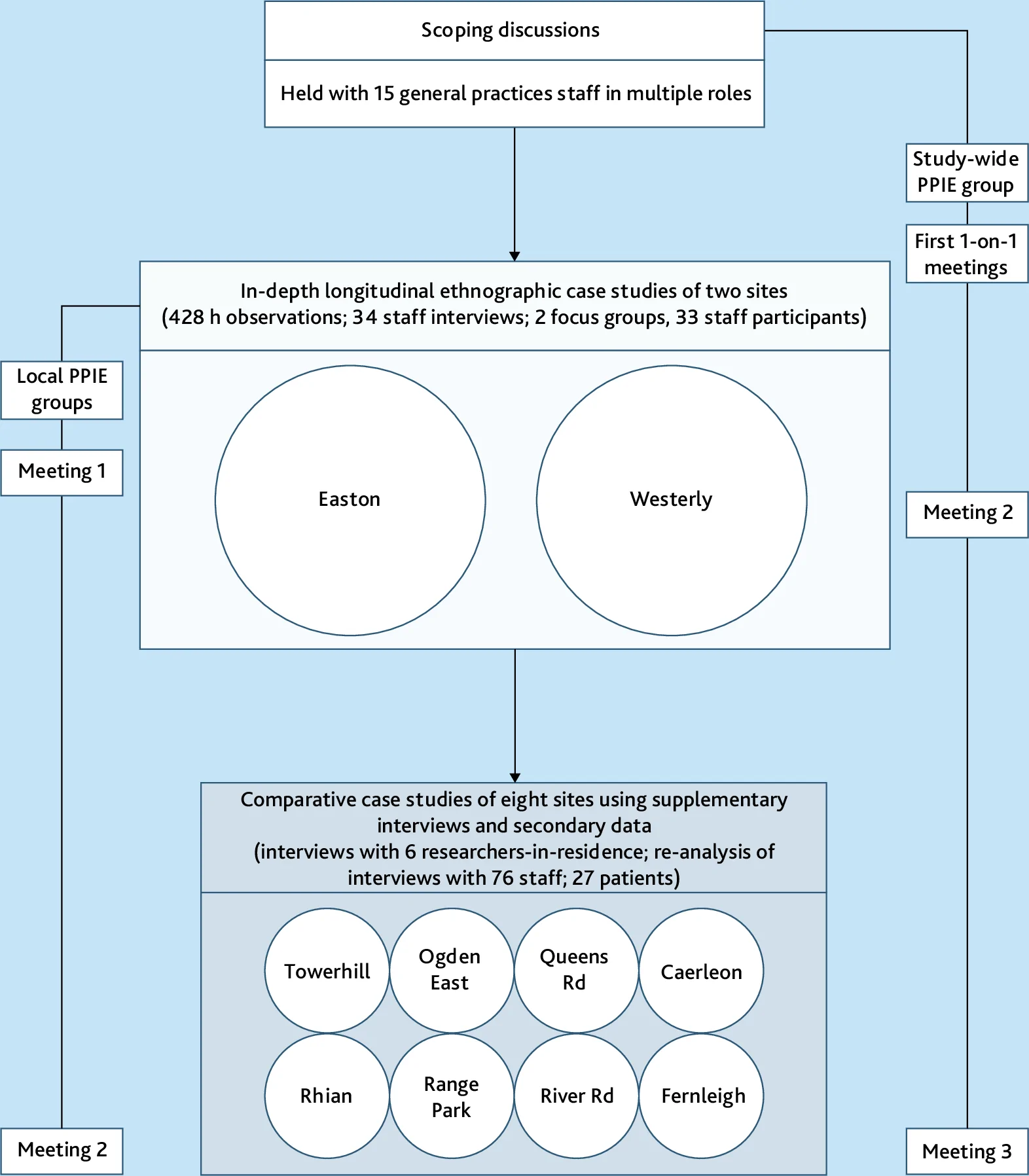
Illustration of the multisited ethnographic case study design. PPIE = patient and participant involvement and engagement. Rd = road.

This design was informed by Eisenhardt’s approach to case study research, which emphasises theory building and explanation through flexible enquiry with multiple cases, empirical data, and theoretical logic.^48,49^ Broadly, this approach uses cases to create theoretical constructs from empirical evidence, utilising a combination of within-case and cross-case analysis.^48–50^ A combination of ethnographic observations, semi-structured interviews (broadly following an interview guide of predetermined open-ended questions), depth interviews (few, open-ended questions),^51^ and focus groups were used. This study design is outlined visually in Figure 1.

The first author conducted extended ethnographic observations in the longitudinal sites, and the third, fourth, fifth, sixth, seventh, and eighth authors conducted sporadic observations at the comparative sites over the study period. The aim of these observations was to close the gap between what people say (or think) and what they do; in this case, ‘work-as-done’ versus ‘work-as-imagined’^52^ to focus on how teamwork happened during change and crisis events. The first author’s longitudinal observations took place in all areas of the practice, including offices, consulting rooms, waiting rooms, and break rooms, rotating between broad teams each week (reception, administration, clinical, and clinical assistant). The first author also conducted depth interviews at the longitudinal sites to discursively explore the impact of digitalisation during the pandemic on participants’ work, teamwork, and wellbeing. This approach enabled participants to share their stories from their own perspectives, focusing on what mattered to them. Interviews typically lasted between 40 and 70 minutes, were conducted in private spaces within the practices or outside (or occasionally online), and were audiorecorded.

The first author conducted semi-structured interviews with the researchers-in-residence at the eight comparative sites, utilising an interview guide developed through engagement with the longitudinal sites and early data analysis. This helped to consolidate data across sites for comparison, and to identify and reflect on how each researcher’s specific lenses or specialty might have influenced what they ‘saw’. These interviews lasted 60–120 minutes, were conducted in private rooms on university premises or online and were audiorecorded. Researchers-in-residence also conducted semi-structured interviews with staff at their named site, typically lasting 30–60 minutes.

Finally, the first author delivered focus groups at the longitudinal sites at the beginning and end of data collection, for a total of four meetings, to discuss the topic of digitalisation during the pandemic with the entire practice team. Each meeting had around eight attendees and lasted between 45 and 60 minutes.

### Patient and participant involvement and engagement

The broader RBD2 programme was informed and overseen by an independent advisory group comprising academics, policymakers, a lay member, clinicians, and a lay chair. Additionally, this substudy involved a multipronged approach to patient and participant involvement and engagement (PPIE). The substudy’s design was informed by scoping discussions with 15 practice staff from across the UK. Thereafter, the study’s progress was monitored by a PPIE steering group of ‘experts-by-experience’, consisting of five general practice staff from clinical, clinical support, administrative, and reception roles, and one patient. The group met three times over the course of the study. The broader RBD2 study was guided by a dedicated PPIE group, who met frequently throughout the project and advised on the early development of this substudy.^44^ Finally, local PPIE was conducted at each of the longitudinal sites to ensure the local appropriateness and acceptability of the study design and focus.

### Data management, analysis, and developing a theoretical framework

The total dataset for the analysis reported on in this article is included in Table 1. Analysis began inductively in the field, with the first author focusing primarily on issues that surfaced empirically rather than strictly following a specific guiding theory or framework.^49,53^ This meant focusing on the field and the data to create first-order and second-order codes;^54^ paying attention to issues that were being spoken about or enacted repeatedly, and making notes on ideas for possible first-order codes in the field, and thereafter reducing that list by using summative or collated second-order codes.^55,56^ Throughout, the first author met regularly with the senior author and researchers-in-residence to review and validate these developments.

**Table 1.** Total dataset

Site number/ pseudonym	Focus group participants, *n*	Observation days, *n*	Staff interviews, *n* (*n* participants)	RIR interviews, *n* (*n* participants)	Patient interviews, *n*
**Longitudinal ethnographic case study sites**					
1 Easton	17	22	18 (18)		0
2 Westerly	16	20	16 (14)		3
**Comparative case study sites**					
3 Towerhill			11 (11)	1 (1)	0
4 Ogden East			8 (8)		5
5 Queens Road			9 (7)	1 (1)	1
6 Carleon			21 (15)		2
7 Rhian			27 (15)	1 (2)	0
8 Range Park			5 (4)		4
9 River Road			12 (10)	1 (1)	0
10 Fernleigh			6 (6)	1 (1)	12
**Total**	33	42	133 (108)	5 (6)	27

Merged cells denote data shared across two sites. Blank cells indicate that data type were not collected for that case study type. A ‘0’ denotes no data collected. RIR = researcher-in-residence.

Once established, these codes enabled a more rapid second level of analysis: thematic analysis using the second-order codes as a codebook in NVivo 1.6.2.^55,56^ To understand the topic of this article, teamwork in change and crisis contexts, the first author looked at first- and second-order codes related to what was or was not helpful to individuals and teams when working amid the changes and crises that were ongoing during our data collection, and presented them to the group for discussion and validation.

First-order examples include ‘asked if I’m okay’, ‘pulling at the same goal’, ‘speak up and ask questions’. Second-order examples included ‘speaking up’, ‘operating knowledge of competencies’, and ‘listen and support’, as outlined in Box 2. It became increasingly clear that interpersonal working relationships were central. Although many of the working conditions and pressures (crises and changes) were similar or the same across case study sites, their impact on wellbeing and efficiency differed because of the relational infrastructures of each organisation, as explored in the results. Throughout these inductive and thematic phases, relevant theory was tested for its relevance and usefulness in discussions among the first author, senior author, and second author, and, where appropriate, incorporated into the analysis. In abductively moving between theory and empirical data, we identified the three key theories discussed in the introduction: relational coordination,^22^ psychological safety,^23,24^ and attentional infrastructures. ^25^

**Box 2.** Examples of grouping first-order codes into second-order codes, and linking theories and concepts

First-order codes	Second-order codes	Linked theory or concept
Everyone feels valued Policy of openness Speak up and ask questions Challenge without aggression No blame	Recognition Speaking up Blame-free	Psychological safety^23^
Pulling at the same goal Trained to work together Pool everyone’s working knowledge We know who’s good at what We don’t know how things are done	Working across teams Working together/teamwork Operating knowledge of competencies	Relational coordination^28^ Teamwork^30^
Making sure nobody is excluded Open-door policy (leader) Everyone does every task (good and bad)	Inclusive leadership Team socialisation (structured)	Advocate (attentional infrastructure)^25^
Somebody will always listen Asked if I’m okay Like a family A big support net They’ll help me Supportive A strong team	Listen and support Practice unity	Team atmosphere Relational support^17^

## Results

This did not begin as a study focused on relationships in teamwork, but rather on the impact of digitalisation on staff work and wellbeing. However, through the process of data collection and inductive analysis, the role of relationality in delivering the complex — and complexifying — work of modern general practice became central. As a result, we searched for theory that could help explain what we were seeing, and abductively testing, extending, or combining them to identify the key elements of a climate of relationality that were most evident in our data. Broadly, staff in practices that exhibited strong relationality across the following elements were better able to navigate the integration of digitalisation during the described change and crisis events; however, there were important caveats to this, which are outlined in the results section. The results section is supported by Table 2 and Box 3, which provide contextual features about the practices (with a focus on digital provisions, Table 2) and describe their team dynamics, Box 3.

**Table 2.** Site characteristics

Site number/pseudonym	Site location	Practice list size	IMD decile	Digital maturityscale point^a^
**Longitudinal ethnographic case study sites**				
1 Easton	South East England	13 500	7th	4
2 Westerly	Greater London	27 000	2nd	3
**Comparative case study sites**				
3 Towerhill	Greater London	15 800	8th	5
4 Ogden East	South West England	8300	1st	1
5 Queens Road	South West England	13 000	7th	3
6 Carleon	North Wales	7500	2nd	1
7 Rhian	South Wales	11 500	3rd	2
8 Range Park	West Central Scotland	2300	1st	1
9 River Road	West Central Scotland	5500	1st	3
10 Fernleigh	South East England	15 000	9th	4

^a^Scale from one to five, where five is the most mature. IMD = Index of Multiple Deprivation.

**Box 3.** Descriptions of technologies and team dynamics at sites

Site number/ pseudonym	Description of technologies used	Description of team dynamics and working conditions
**Longitudinal ethnographic case study sites**		
1 Easton	Many patients use remote access routes. GPs often used telephone consultations, occasionally used photo-sharing software, and none made use of video consulting	The workforce had strong retention, with practice staff being very familiar with one another and referring to the organisation as ‘a family’. Staff were embedded in the local community with a strong awareness of individual patient circumstances and needs
2 Westerly	Offers access via telephone, website, and NHS App, but had mixed uptake and is re-introducing a walk-in-and-wait service. Very frequent user of the digital interpretation service LanguageLine. Telephone contact is very high, placing a significant burden on reception staff and driving high turnover rates	Clear divide between support and clinical staff teams, and a hierarchical staffing structure that favours the practice manager and partners’ voices. Relationships within the practice have suffered since 2020, with more clinical and senior staff able to work remotely, and support staff having little control over how, when, and where they work owing to infection control measures and new ways of working. High turnover rates, particularly in reception roles. Clinical staff have better retention but often discuss moving on. Low value placed on staff health and wellbeing
**Comparative case study sites**		
3 Towerhill	Access is officially digital-only, with a high proportion of consultations occurring remotely. Clinical staff support the digitalised service, but support staff identify the risk of digital exclusion and manually add telephone or walk-in patients to the digital queue to avoid them being pushed back	Clear divide between the clinical and support staff, whereby clinicians were viewed as the ‘centre’ of the practice, and support staff as peripheral and transient. Clinicians have a supportive environment with good communication, although less social cohesion and increased isolation since going fully digital. Suggestions that this is leading to increasing clinical turnover. Support staff viewed as external, not central to the practice. Reception and admin teams have low support, lack a team atmosphere, and have high turnover rates
4 Ogden East	Some online consultations offered, but aware of the risk of digital exclusion. Remote consulting generates a higher workload than in-person options, and remote triage is not used	Focused on maintaining ‘traditional’ general practice embedded in their population’s needs: continuity, support, familiar faces. Staff take a team approach to problem-solving. Strong retention of all staff, GP registrar tend to stay on after training
5 Queens Rd	Offers online, telephone, and walk-in access, and the practice has a very high uptake of Language Line. Staff experienced some patient abuse in response to digitalising access services	Decisions were partner-led, with less focus given to the views of salaried staff (clinical and support). Siloed and isolated working. Staff get frustrated by technology not functioning, reflected in their interactions with one another. Little capacity to feed back to leadership, and no value placed on staff wellbeing. Higher turnover
6 Carleon	Offers only phone or walk-in contact, largely owing to staff and patient preferences	Staff are loyal to the practice, a ‘family’ who all know each other. Culture of speaking up, with value placed on all staff
7 Rhian	Telephone triage and some telephone appointments are available, but no online access or consultations yet	Staff rarely change and have worked together for a long time, meaning strong teamworking culture. Retains GP registrars. Collaborative and communicative across the whole practice. Relational hierarchy: decisions made collaboratively by practice manager and partners, in open consultation with other staff
8 Range Park	Mostly traditional, in-person care. Some consultations are done over the phone, with practice interest lying more in community-driven development than technological	The practice has a ‘family’ ethos; the workforce is very small and embedded in the community, with strong familiarity and trust between staff members and between staff and patients. Relational hierarchy: clear distinctions between partners and other employees, but clear communication routes and strong focus on respect across these power divisions
9 River Road	Online triage recently introduced. Staff responses mixed, with a higher burden on GPs to triage e-consults to reduce strain on reception	Close-knit team with strong camaraderie. Team works collectively to meet the demands of the practice daily, with a clear culture of trust in one another to perform their share of workload. Open communication between individuals and teams
10 Fernleigh	Embraces digitalisation where possible, and actively adapts work processes, although many patients are older adults and do not engage with online access routes	Culture of psychological safety and speaking up, approaching mistakes as an opportunity for learning. Value different kinds of knowledge from all staff groups. However, leaders struggle to make space for building relational links between staff (for example, through breaks) owing to supply and demand gap.

We structure our results by highlighting different elements of relational climates, using terms and concepts derived from the theories in our framework; for example, relational advocacy from Bartel and Rockmann,^25^ speaking up and psychological safety from Edmondson *et al*,^24^ training for teamwork, relational job design, and team meetings from Gittell.^22^ Other themes we constructed from the data in conversation with theory (for example, relational leadership, relational support, the dark side of relationality). We support these findings with quotes from field notes and interviews with staff and researchers-in-residence.

### Relational infrastructure

The practices included in this study differed in the extent to which staff felt part of a team and well supported by the local relational infrastructure to work in that team. In many of our practices (Easton, Fernleigh, River Road, Range Park, Rhian, Carleon, Ogden East), the existing relational infrastructure was stronger. This was evidenced by the way that staff spoke about (and to) one another, and in how they behaved around one another. Staff talked about these organisations as being a ‘strong team’, ‘family’, ‘a social net’, who were ‘in the same boat’ during change and crisis events. This helped to orient staff towards ‘the same goal’, support each other, and improve retention during times of change and crisis, as described in the following quotes:

*‘The ethos here is like a big social net, and we all support one another.’* (S2S9, interview with phlebotomist–receptionist, Easton)

*‘Staff turnover in both places wasn’t high. There was a certain loyalty about it. They’d been there long enough they felt like family. They knew everybody was on the same boat.’* (S6S7RIR-A, interview with researcher-in-residence, Carleon and Rhian)

*‘You feel like you’re pulling at the same goal together.’* (S10RIR, interview with GP partner, Fernleigh)

In these practices, staff tended to acknowledge the value of each member of the team, contributing to a collective strength. Often this would involve actively redistributing workloads to ensure no one individual was overburdened. It was also made clear that this team-making was an active practice built together through recognising and acknowledging everyone’s competencies, commitment, shared workload, and mutual respect:

*‘We have a strong team; we all bring different things to it. We all have a commitment to the job, commitment to each other, and respect for each other.’* (S2S15, interview with GP partner, Easton)

*‘In Carleon, the doctors, they supported each other. If someone was pulled somewhere, the others would take over. Same in Rhian, the salaried doctors described how the reason they liked it was because of the teamwork, and they will share the workload.’* (S6S7RIR-B, interview with researcher-in-residence, Carleon and Rhian)

Successes would be framed as collective achievements rather than individual, as described by the following quote from a phlebotomist–receptionist:

*‘I'm glad that he’s got the help. You can't think I did that* [alone] *because we all did it; we each do part of it* […] *We respect each other. The whole building works as one team.’* (S2S9, interview with phlebotomist–receptionist, Easton)

In other practices (primarily Westerly, Queens Road), our data suggested that the relational infrastructure was less strong — ‘relationally indifferent’, or ‘antipathic’. In these cases, staff cohesion and wellbeing were worse. This was evident in the way staff spoke about one another and about their work, and in defensive responses to workload. For example, some reception staff in Westerly expressed suspicion of those working remotely, implying an uneven workload distribution that escalated perceived pressure in these roles:

*‘It’s 9 a.m. Reception is sparse today. The reception team lead and the receptionist are the only ones in. “I don’t mean to moan but it’s a bit much for two people to handle mate. It’s the same people over and over again going off to work at home, and it’s the people in charge who get paid twice as much as us. I’m meant to be out front* [on the desk] *but there’s too many e-consultations.”’* (S1S6, reception team lead [Fieldnotes from observations at Westerly])

In one practice, Towerhill, we observed a clash in the coherence of the local relational infrastructure, whereby the clinical team experienced a strong relational infrastructure, were well supported, and felt part of a team, whereas in the reception and administrative team the opposite was true. This disconnect was heightened by the hierarchical nature of the organisation’s structure:

*‘There were two layers to the hierarchy. One with the clinicians on the top. And the lower level, that’s the admin and reception. In admin it’s a bit less supportive, there’s less of a team feeling, less connected — they’re not seen as the core of the practice.’* (S3RIR, interview with researcher-in-residence, Towerhill)

### Actions contributing to relational infrastructure

In this subsection we describe actions and approaches that contributed to stronger relational infrastructure, improving teamwork and staff wellbeing.

#### The actions of leaders: relational leadership

In practices where team relations were positive and supportive, there was evidence that those in formal leadership positions (GP partners, practice manager, team lead), and those in social leadership positions (longstanding staff) were taking steps to enable and nurture relationships among staff, and acted in the interest of staff wellbeing.

Formal leaders practised relational leadership in a variety of ways. For example, in the Towerhill clinical team, a partner established an ‘open-door policy’ to encourage friendlier interactions with trainees and physician associates:

*‘We’ve got an open-door policy at the surgery, we’ve got numerous trainees and PAs* [physician associates] *and they can come and ask me questions, I’m probably the most friendly so they generally tend to knock on my door.’* (S3NB, interview with GP partner, Towerhill)

In other practices, leaders actively encouraged psychological safety through establishing a speaking-up culture, fostering relationships through frequent communication and peer learning:

*‘If you can have a culture where it’s not a problem at all for you to just ask a question, you can have a good relationship.’* (S10RIR, interview with GP partner, Fernleigh)

*‘One of our aims is to make everyone feel valued in the practice* […] *We have an open policy so that everybody can say “this isn’t working” or “I think this would be better if we did it this way’’.’* (S6CHK, interview with salaried GP, Carleon)

This leadership style translated to feelings of support and respect, helping to build a ‘team’ atmosphere. The following quotes, one from a dual-role phlebotomist–receptionist who had been in-post for over a decade and the other from a recently qualified GP, exemplify the impact of relational leadership on the daily experiences of staff across roles and regardless of time in-post:

*‘The doctors have got so much respect for us. We can have a laugh and also be serious. It’s an additional family. I could go and knock on any one of their doors. It’s really supportive. You’re not separated even though you have different jobs.’* (S2S9, interview with phlebotomist–receptionist, Easton)

*‘There’s support when we need it. That’s the reason I stayed here. Everybody chips in.’* (S7ZT26, interview with recently-qualified salaried GP, Rhian)

#### Team actions: open communication routes and speaking up

In practices with open communication routes, we saw that issues could be discussed and resolved clearly and quickly. This could occur formally or informally, but communication routes were maintained through frequent use. Empirical instances of these in more relational practices — such as direct messaging, open-door policies, and ‘go-to’ personnel — are described here:

*‘If reception had an urgent question they would use the EMIS messaging system, but trainees would go in and ask GPs questions in their room. It was a very collegial environment.’* (S8S9RIR, interview with researcher-in-residence, River Road)

*‘In Rhian there were specific communication pathways. Everyone had a go-to person, for clinical queries and pastoral needs, and they may not be the same person. If they were a receptionist, they would go to the manager, assistant manager, or the head receptionist.’* (S6S7RIR-A, interview with researcher-in-residence, Carleon and Rhian)

In more relational practices, these open communication routes facilitated speaking up, a term from psychological safety describing how staff might raise concerns when things go wrong. For example, the ‘near-miss’ incident described in the next quote involved speaking up across a hierarchy to prevent harm to a patient, the successful avoidance of which is framed as a collective achievement:

*‘There was a gentleman that I know on the phone to another receptionist. She said it wasn't urgent. But I know he never phones, that was a red flag to me straight away. So I said, quietly “he needs to go on the urgent list”*. *One of the nurse practitioners recognised him and said it was probably just his drinking, he’s an alcoholic. I said, “I know what he is. But you haven't seen him”. I was polite but I had to challenge it. She asked for me to do him an urgent blood test instead of a nurse appointment. So we booked that. When he came in I went straight to the same nurse and I said, you better come see him; his nose is black his legs are swollen. She done him an ECG. He’s only having a heart attack. I knew there was something wrong because **he called**.’* (S2S9, interview with phlebotomist–receptionist, Easton)

Similarly, a GP partner reflects on the benefits of speaking up when under times of high pressure. When staff felt safe to speak up about feelings of anxiety, being overwhelmed, or uncertainty, it translated to a more secure and supported experience of work:

*‘When you are overwhelmed, people help. And I’ve worked in places where that is not the case. So if you put your hand up and say “I can’t cope”; it would be done without me having to ask again. And that’s really valuable.’* (S2S15, interview with GP partner, Easton)

#### Team actions: relational support between staff

In more relational practices, staff often sought to support one another in times of high pressure or personal strain, which improved endurance. For example, in the instance in the next quote, a receptionist had a challenging interaction with a patient but was supported by their direct colleagues to navigate their emotions during those moments:

*‘I did only break down crying once and* [my colleague] *said just go, put the phone down. She said just go and make yourself a cup of tea, come back in 10 minutes, and that 10 minutes was all I needed.’* (S10BH19, interview with receptionist, Fernleigh)

General practices that did not foster relational support tended to have fewer opportunities to release built-up pressure, create relational bonds, or raise concerns, as described here:

*‘There’s less opportunity to really feedback anything about what’s going wrong, if you're having difficulties at all. But I don't know that there’s an informal way to just really have a good moan about your workload. I think that’s missing.’* (S4S5RIR, interview with researcher-in-residence, Queens Road)

Less relational practices tended towards apparent ‘quick fixes’ in response to staff wellbeing problems, such as directing staff to wellbeing programmes or NHS staff therapy. However, these offers could be contradicted when the organisation itself was relationally inconsistent in the design of its working day; for example, misalignments between support that was ‘on offer’ and time available within a working day to access that support. This highlighted the lack of reciprocity in boundary blurring — whereby staff often allowed ‘work time’ to spill into ‘home time’, but no activity beyond work tasks could enter into a working day. Such internal contradictions drove frustration in staff and risked further harm, as described in the two examples here:

*‘Those wellbeing programmes don't really work because we work. I tried to do one before, and I couldn't do it because I couldn't get the time off work. They're in working hours.’* (S1S5, interview with reception team lead, Westerly)

*‘The PCN* [primary care network] *started providing* [staff] *with mental health support therapy, which I used. And I got a note to file from HR* [human resources] *to say this is fraudulent behaviour, using therapy during work hours.’* (S1S7, interview with clinical assistant, Westerly)

### Organisational routines contributing to relational infrastructure

In this subsection, we describe the broader organisational routines that we saw as contributing to relational infrastructures.

#### Training for teamwork and relational job design

Some practices adopted a team-centred approach to work, embedding it within local training approaches. This involved training staff members to view their work as a collaborative effort, highlighting task interdependencies, and improving awareness of one another’s roles and skills. This improved staff understanding of how to work together and shared responsibilities, and fostered a shared dedication to their work. When present, this training style improved coordination of work, cohesion around the identity of the ‘team’, and encouraged a relational approach to working, as described in the following excerpts:

*‘Understanding* [each other’s roles] *is important. I think it allows you a better understanding of what their thinking was when they did something. It allows you to interface more effectively with them so that you’re not duplicating or replicating what they're doing. You can have a much more efficient thing.’* (S10RIR, interview with researcher-in-residence and GP partner, Fernleigh)

In some practices in our sample, this training took place formally (through induction and formal training workshops), whereas, in others, it occurred informally (through social interactions and ongoing team-centred behaviours by individuals). Staff in practices with a more team-centred approach to training reflected on how this contributed to the practices’ retention of staff, as described by this practice manager:

*‘Quite a few of our salaried GPs are GPs who trained here and have stayed, and that says something about their intention, their dedication. It says something about their trainers* […] *this practice genuinely is dedicated to its patients.’* (S4BT2, interview with practice manager, Ogden East)

Our data indicatethat team-centred training in such practices contributed to improved retention, as the following GP describes:

*‘How we’re trained matters. It can make you narcissistic, or that kind of person where you’re dog-eat-dog to get training posts. It’s also who you work with. We both trained here and stayed. Same with all the partners. It’s always a good sign when the trainees stay on at the surgery.’* (S7ZT26, interview with recently-qualified salaried GP, Rhian)

This, in turn, helped with ensuring the continuity of team-centred staff training into the future, whereby established members of staff passed on their knowledge and approach to new starters, as a partner from Fernleigh describes:

*‘A load of our staff are trained through interactions with longer-term staff and the sort of tacit knowledge that they have rather than protocols and processes. I just think you can’t quantify the value that those long-term staff bring.’* (S10RIR, interview with researcher-in-residence and GP partner, Fernleigh)

In practices where there was a lack of team-centred training, staff identified that there were gaps in their understanding of the whole system, their role in it, how specific workflows passed between teams, and where interdependencies were. This inhibited relational coordination and impeded work, as described in this quote:

*‘The* [e-consult] *requests are being triaged by a doctor, but they need to be actioned by one of the receptionists. But there isn’t the training on the system. If reception were shown a bit more of how the system works or the practice works, I don’t think the turnover would be as much.’* (S3GF2, interview with patient liaison officer, Towerhill)

#### Shared (work)spaces

The more relational practices in our sample had physical or digital spaces where staff were likely to interact, which enabled relationship formation, knowledge exchange, and improved coordination. This type of interaction commonly occurred in shared workspaces (such as an open-plan reception office) or shared recreational areas (such as break rooms). Shared reception spaces, for example, improved relational coordination for reception and clinical teams, as most members of the multidisciplinary team would pass through this space mutliple times a day, offering opportunity for ‘cross-working’ and communication:

*‘Rhian’s reception would constantly have admin people or clinical people coming to reception, dropping things off, asking things. A lot of cross-working there.’* (S6S7RIR-A, interview with researcher-in-residence, Caroleon and Rhian)

Sometimes, these spaces could be digital — such as formalised communication software run on practice computers (like Microsoft Teams) or through informal app-based group messaging platforms (like WhatsApp). These (digital and physical) spaces provided an opportunity for more immediate input into work tasks, and overcame distances in remote teams, offering improvements to coordination and efficiency as described in the next quote. However, these benefits came alongside the risk of workflow interruptions and work–life boundary blurring:

*‘[In Carleon], they had this WhatsApp group where the pharmacist would write a request, the GPs would write to him and then the receptionist would see it. It was on a phone in reception. They would always check that phone to see if the pharmacist had written. It was very slick. Even though it was remote, he felt very supported.’* (S6S7RIR-A, interview with researcher-in-residence, Carleon and Rhian)

Practices with workflows that did not move as clearly into other teams’ physical spaces instead maintained places where staff would cross paths with one another at certain times of the day, like the staff room:

*‘People tended not to disrupt one another because they knew at some point they’d come and get coffee in the staff room. It was a key space.’* (S8S9RIR, interview with researcher-in-residence, River Road)

In practices where shared space was lacking, and staff were more isolated by their working routines, relationships and coordination suffered. In these organisations, there was less opportunity for relationship building, social interaction, and knowledge transference. Without these socialised pauses in working days, staff also experienced lowered morale, burnout, and isolation, and found it difficult to seek support, as described by the partner here:

*‘I sit in my chair about 5 hours every morning and then 5 hours in the afternoon* […] *We’re losing that cohesion within the surgery. Doctor retention and nurse retention are reduced if you can’t interact with your colleagues; you can’t ask advice and speak to each other as you would normally, that normal camaraderie you get in fighting the workload.’* (S3NB, interview with GP partner, Towerhill)

#### Team meetings

In our dataset, regular team meetings provided an opportunity to reinforce relational links between staff members and improve understanding of role/task interdependencies. This is likely owing to these occurring in practices where there was existing relational infrastructure as described above. In such practices, team meetings focused on both professional and pastoral concerns, helping staff to seek support for the effects of change and crisis in the workplace. One practice’s daily ‘coffee morning’ included representatives from all teams (reception, administration, leadership, nursing, GPs) and functioned as an opportunity for individuals rostered on that day to socially catch up and to collectively run through the emergency triage list, sharing their pooled knowledge of the patients therein, as described:

*‘Our emergency list gets triaged every day, not just by one clinician, but by multiple during clinicians’ coffee. It is a chance to sit down with all the clinicians and me and the head of reception to talk through everybody that’s on the triage list.’* (S2S3 interview with practice manager, Easton)

In particular, in-person team meetings could be focused on resolving problems, which could be hard to achieve in an increasingly digitalised and isolated working environment. These meetings allowed staff the opportunity to speak up about problems they were facing and seek help:

*‘If you’re meeting with people on a regular basis, and you're saying, “I’m really not coping at the moment”, talking about the problems, then it can get sorted out relatively quickly and in a quite informal way.’* (S4S5RIR, interview with researcher-in-residence, Ogden East)

In our dataset, practices that did not have whole practice meetings (or only rarely hosted smaller team meetings) were also less relational. The absence of these routinised opportunities to socialise, share successes, and share problems was felt by staff, as described in this quote:

*‘More practice meetings would be excellent for everybody to get to know one another. Everybody putting in their concerns, positive feedback, negative feedback. I’ve been here for 3 years, we’ve had one practice meeting. It’d just be nice to just get to know everybody and be working from the same sheet to make the practice better.’* (S1S6, interview with receptionist, Westerly)

### The dark side of relationality

It is important to note that the cross-case analysis also indicates negative aspects of relationality in GP practice teams. First, we observed that, where team cohesion was strong, it could highlight individuals who did not ‘gel’ with other staff members. In our data, reasons cited for this included personality clashes, differences in perceived values, and practices that explicitly valued clinical roles over reception. When present, this was experienced negatively by affected staff. Second, given the distribution of the whole practice team across multiple subteams, conflicts could arise between the relationality of one team and another. We saw this conflict occur in one practice’s reception and clinical teams, which divided the practice and damaged coordination and communication across subteams, as described here:

*‘*[Reception staff] *sometimes interact* [with clinical staff] *but not on a regular basis. We tend to only interact when it comes to a patient query or if* [a] *doctor’s walking by and we’ve actually got a minute to have a conversation, but generally we don’t really mingle with the clinical staff.’* (S3GF2, interview with patient liaison officer, Towerhill)

Third, staff in highly relational practices who felt loyalty towards their fellow team members tended to overstretch themselves to support each other during times of crisis and change, contributing to burnout:

*‘General practice is death by a million paper cuts. When you’re always firefighting, and you’re always running. It’s really hard to stand still, and create the space to step out of it, stop and take stock.’* (S2S15, interview with GP partner, Easton)

*‘In Carleon, the partners were more protective of the other staff, so extra work would come to them because they felt like it was their problem, so their work was overflowing. They had a non-stopping list.’* (S6S7RIR-B, interview with researcher-in-residence, Carleon and Rhian)

## Discussion

### Summary

In this paper, we report on a subanalysis of data collected for a larger study on technology integration in UK general practice, focusing on how teamwork is affected by change and crisis contexts that unfolded between 2021 and 2024. Through applying relational theories from organisation studies, including relational coordination, psychological safety, and attentional infrastructures, we found that a strong relational infrastructure is desirable in general practices as it contributes to a stronger team identity and improved teamwork, relational coordination, and staff wellbeing, particularly in the face of change and crisis events. However, we also outline the ‘dark side’ of relationality; specifically, its exclusionary capacity and the burden of ‘relational maintenance’ on team members and leaders. In our results section, we outline individual-level actions and organisational-level routines that reinforced the relational infrastructure. In Box 4, we summarise these actions and routines as they appeared in our data, suggesting ways that practices could approach building relationality within their own teams.

Box 4. **Individual actions and organisational routines reinforcing relational infrastructure**

**Individual actions reinforcing relational infrastructure**

- Relational leadership: leaders actively attending to and enabling working relationships between team members and across teams. Leading by example, creating opportunity for collaborative working and open communication, facilitating staff psychological safety to speak up
- Open communication routes: create formal and informal communication routes, for example, clear pathways for communicating about common issues, designate contact people for each team, set up messaging platforms for whole-practice teams, set up ‘office hours’ or an open-door policy
- Speaking up: ensure that any concerns or issues raised by any member of staff are treated with equal respect and with a learning mentality (as opposed to blame), to encourage staff to feel psychologically safe to speak up within and between teams
- Relational support between staff: encourage staff to support one another informally through taking shared breaks, discussing shared challenges, and opportunities to decompress during periods of high stress

**Organisational routines reinforcing relational infrastructure**

- Team-centred training and work: engage in team-based training whereby trainees learn from (and with) their real colleagues, engage in peer-to-peer learning on the job, and are encouraged to ask questions
- Shared (work)spaces: provide spaces where workflows naturally overlap to allow for communication and coordination as work is done, create dedicated non-working spaces, such as a breakroom, where staff can interact informally
- Team meetings: ensure that teams are having regular meetings with one another at the level of their roles (such as clinical and support staff), as well as whole-practice team meetings, to ensure that all groups can communicate with one another confidently and understand one another’s’ roles and their intersections and interdependencies
- Regular check-ins: build in regular check-ins with team members to ensure that relationality is not enabling overworking and risking burnout — this can be done between team leads and team members, and between members of the leadership team

The period under study incorporated a uniquely tumultuous period in healthcare settings and in society more broadly because of the COVID-19 pandemic. As described in the introduction, this backdrop and its associated impact on primary care workforce numbers,^4,13^ workload,^11,15–17^ mental and physical health,^6–10,18^ and reputation created especially fraught conditions for studying change and crisis.^12^ This may have made the contrast between more and less relational organisations more stark, thereby more clearly highlighting their benefits. What was clear through our extended engagement with these general practices was that these organisations considered themselves to be working under ‘firefighting’ conditions both before and after the ‘acute’ phase of the pandemic (2020–2023, the period under the World Health Organization Public Health Emergency of International Concern status).^57^ As such, change and crisis can be considered constant features of working in UK general practice, and staff must continually adapt to this and weather the impact on their wellbeing to achieve daily work.

Therefore, these results have strong relevance to practice and policy in understanding the local relational conditions that support overcoming cumulative crises affecting everyday work, and in strengthening team preparedness for abrupt crisis events, such as the pandemic. In general practices that were relationally antipathic or indifferent, staff had to learn to adapt in isolation and were unlikely to receive meaningful or practically actionable support in protecting their mental and emotional wellbeing. Practices that were advocates of relationships and enabled individual and organisational routines and practices for improved relational coordination and psychological safety tended to better navigate change and crisis events through collaborative efforts, open communication, and speaking up. Staff in these organisations were better able to seek and provide relational support from one another, thereby reducing the ill effects of change and crisis on their wellbeing.

However, building and sustaining relational infrastructure is a complex and intensive process, requiring constant attention from leaders and other staff. In a broader landscape of workforce shortages, funding challenges, and policy constraints, building a relational infrastructure may be difficult to prioritise and the process by which to do it opaque. We hope that Box 2 will enable staff and practice leaders to identify elements that could be enhanced or integrated in their own organisations; however, we share it in the full knowledge that further work is needed to establish how best to embed these actions as routine.

In conclusion, this paper offers the term ‘relational infrastructure’ to describe the bundle of organisational routines, processes, and interactions that enable staff to feel stronger team coherence and unity, communicate more effectively, and coordinate their work better. Our article has evidenced that, where relational infrastructures are stronger, practice teams have improved unity, have better coordination and communication, and are more able to weather the challenges of working through constant change and crisis events. Fundamentally, we identify that working relationships are a critical infrastructure in achieving the complex, technologically mediated work of modern general practice in the UK.

### Strengths and limitations

This study used a robust and extensive model of case study research that generated rich data from ethnographic observations and in-depth interviews from general practices across England, Wales, and Scotland. Unfortunately, no practices in Northern Ireland or the north of England took part; therefore, more research is needed to determine the relevance of our findings in these locations. Our use of the researcher-in-residence model facilitated deep engagement across sites and generated excellent insights into the local organisation’s dynamics, routines, and atmosphere; however, it is worth noting that all researchers have their own lenses and approaches to research, which will have informed their interpretations of the sites.

### Comparison with existing literature

The value of relational continuity has long been discussed in the context of clinician–patient relationships and patient outcomes in UK general practice. There is a robust and growing evidence base supporting its multiple benefits (on patient experience, outcomes, and staff workload),^40–43^ as well as the requirements for achieving different kinds of continuity in modern general practice.^58,59^ The results presented in this article indicate that we could extend our conceptualisation of relational continuity to consider the importance of relationships between practice staff in both creating better working conditions for staff *and* delivering high-quality care to patients. These relationships are encouraged by stronger relational infrastructures within the GP practice as an organisation, which we framed through the concepts of relational coordination, psychological safety, and attentional infrastructure.

We previously identified the value of relational coordination in enabling the complex and interdependent work of technology-enabled GP practice work,^17^ and we further developed its relevance in UK general practice by integrating the theory into our conceptualisation of ‘relational infrastructure’. By applying it as part of our theoretical framework and abductive analysis, we have also demonstrated its qualitative utility, extending its application beyond the survey-based methodological approach often associated with relational coordination.

Psychological safety has been successfully applied to UK primary and secondary care settings,^36^ and healthcare contexts internationally.^34,35^ Research grounded in UK general practice identified specific barriers (for example, hierarchy, perceptions of knowledge, and authoritarian leadership) and facilitators (for example, leader inclusiveness, open culture, boundary-spanner roles, and strong interpersonal relationships) to achieving psychological safety.^60^ Our results indicate that we must move beyond barriers and facilitators, and approach psychological safety in general practice as part of a broader relational infrastructure that is continually (re)established and negotiated through the daily actions — or inactions — of individual staff. We also argue that criticisms of hierarchy must be refined to identify the *approach* to hierarchy (for example, relational versus antipathic) as critical, given that medical settings are (by necessity) stratified environments. The final element of our conceptualisation of ‘relational infrastructure’, attentional infrastructure, has yet to be applied in healthcare contexts, to the authors’ knowledge; therefore, this constitutes a novel contribution and highlights the value of interdisciplinary perspectives in health services research.

### Implications for research and practice

Our work demonstrates the staff- and organisational-level benefits of nurturing relational infrastructures in UK general practice and provides some guidance on specific actions and routines that can support this (while being aware of the associated burdens), for use by practices. These infrastructures should be acknowledged as critical in improving staff wellbeing, coordination of work, and therefore contributing to better service delivery for patients.

We would encourage policymakers to attend to the evidenced value of more relationally grounded working contexts in general practice, particularly given their capacity to encourage staff retention. Box 4’s individual actions and organisational routines for relational infrastructures should provide guidance in terms of areas to focus policy efforts and funding on, but we consider specific areas for policy consideration to include:

- including a specific focus on team-centred training and relational leadership training in future training policy or ring-fenced training funds;
- establishing guidance on structuring practice working days to ensure cross-working and relationship-building opportunities arise (for example, shared break times and regular intra- and interteam meetings); and
- more broadly, centring on the value of strong working relationships in primary care.

Learning and embedding the value of these relational infrastructures is crucial in creating a working environment where staff can feel well supported to deliver their daily work and navigate future change or crisis situations. This is particularly important now, as the service must adapt to policy goals of further digital transformation, as outlined in the new 10 Year Health Plan.^61^

Having identified the benefits of relational infrastructures where they already exist, future research should attend to how these relational infrastructures can be constructed, sustained, and evaluated over time. Alongside this, research should attend to the coexisting negative impact that relationality in organisations can contribute to, and how to overcome this.
